# Validating Accuracy of a Mobile Application against Food Frequency Questionnaire on Key Nutrients with Modern Diets for mHealth Era

**DOI:** 10.3390/nu14030537

**Published:** 2022-01-26

**Authors:** Joyce D. Kusuma, Hsiao-Ling Yang, Ya-Ling Yang, Zhao-Feng Chen, Shyang-Yun Pamela Koong Shiao

**Affiliations:** 1 Heritage Victor Valley Medical Group, Big Bear Lake, CA 92315, USA; 2 Mt San Antonio Gardens, Pomona, CA 91767, USA; 3 School of Nursing, College of Medicine, National Taiwan University, Taipei 10051, Taiwan; slyang@ntu.edu.tw (H.-L.Y.); ylyang@ntu.edu.tw (Y.-L.Y.); 4 Department of Nursing, Yuanpei University of Medical Technology, Hsinchu 30015, Taiwan; ivan.chen1966@gmail.com; 5 Center for Biotechnology and Genomic Medicine, Medical College of Georgia, Augusta University, Augusta, GA 30912, USA

**Keywords:** personalized nutrition, mobile applications (mobile app), Food Frequency Questionnaire (FFQ), modern diets, generalized regression, mHealth, dietary record, agreement and bias

## Abstract

In preparation for personalized nutrition, an accurate assessment of dietary intakes on key essential nutrients using smartphones can help promote health and reduce health risks across vulnerable populations. We, therefore, validated the accuracy of a mobile application (app) against Food Frequency Questionnaire (FFQ) using artificial intelligence (AI) machine-learning-based analytics, assessing key macro- and micro-nutrients across various modern diets. We first used Bland and Altman analysis to identify and visualize the differences between the two measures. We then applied AI-based analytics to enhance prediction accuracy, including generalized regression to identify factors that contributed to the differences between the two measures. The mobile app underestimated most macro- and micro-nutrients compared to FFQ (ranges: −5% for total calories, −19% for cobalamin, −33% for vitamin E). The average correlations between the two measures were 0.87 for macro-nutrients and 0.84 for micro-nutrients. Factors that contributed to the differences between the two measures using total calories as an example, included caloric range (1000–2000 versus others), carbohydrate, and protein; for cobalamin, included caloric range, protein, and Chinese diet. Future studies are needed to validate actual intakes and reporting of various diets, and to examine the accuracy of mobile App. Thus, a mobile app can be used to support personalized nutrition in the mHealth era, considering adjustments with sources that could contribute to the inaccurate estimates of nutrients.

## 1. Introduction

A dietary intake of key essential nutrients is important to promote and maintain human health and to reduce health risks across the life span, which can be achieved with a personalized diet [1,2,3,4,5,6]; however, significant proportions of the population do not consume the recommended daily intake of essential nutrients [7,8]. Inadequate intake of essential macro- and micro-nutrients can lead to mortality, morbidity, and create a burden on healthcare systems [1,2,3,4,5,6]. With the accessibility of modern smartphones, the essential micro-nutrients can be assessed conveniently through internet-based health applications (apps) to enhance healthy nutrigenomics and epigenetics [9,10,11]. In addition to energy-producing macro-nutrients (energy nutrients) of carbohydrates, protein, and fats [12,13], micro-nutrients such as folate (B9), cobalamin (B12), choline, and amino acids, including methionine and glycine, are methyl donors essential for DNA methylation in nutrigenomics pathways [4,14,15,16,17]. Hence, accurate measurement tools in assessing macro- and micro-nutrients are critical for personalized nutrition to improve health outcomes across vulnerable populations [18,19].

The Food Frequency Questionnaire (FFQ) has been comprehensively used to examine the associations between diets and disease risks across populations in many countries with its variations of diets [19,20,21,22,23]. The FFQ includes the frequency and portion size of foods and beverages consumed over time (30 days to 12 months) with additional items to adjust fat intakes from various sources [22,24,25,26]. FFQ is advantageous for its low-cost, relative ease of use, ability to measure dietary intake over time, and ability to measure nutrition consumption in large populations [19,21,26]. FFQ was validated as a dietary measure [19,20,21,22,23,24], against 1-day [21], 2-day [23,24], 3-day [20,22], and 6-day [19] dietary records with the caloric ranges of 500–5000 [27,28], in association with disease risk (506 colorectal cancer patients and 673 controls aged 20–74 years) [27]. As an example, FFQ was validated against a 4-day weighted food record with a convenient sample (female participants aged 16–45 years of Māori, Pacific, or European ethnicity); therefore, this information might not be generalized to other populations [28]. The FFQ presented greater estimates on macro- and micro-nutrients compared to the 3–4 day diet records on macro-nutrients (10–20%) and micro-nutrients (10–30%), particularly fat-soluble Vitamin E (15–30%) and Vitamin A (>50%) [28,29]; people who might consume extreme ranges of calories, <500 and >3500 calories per day, were cautioned for possible inaccurate reporting on dietary intakes [4,12,22,30].

There are numerous diets across the world in the modern era. The culture, climate, soil characteristics, and socio-economic status have nurtured the various traditional diets, including examples in the Mediterranean, Italian, convenient American, Chinese, or Japanese diets [31,32]. With urbanization, globalization, and the aging population, convenient fast foods [33,34], high school foods [8], smoothies [35,36,37,38], liquids [35,39], and dozens of other diet types have been influenced by immigration [31]; however, many diets are reported with insufficient essential nutrients; many Western diets could contain excessive empty calories [40] and rice-based Eastern diets would have insufficient zinc and iron [41]. Accurate assessment of nutrient intakes and more conveniently accessible tools such as mobile apps are critical for personalized nutrition [42].

Bland–Altman plots and correlation coefficients are commonly used univariate analytics to verify the accuracy of a new measure against another established measure [43,44]. The Bland–Altman plots illustrate the % differences between the two measures, where variability increases with increased differences [43,45,46,47]. The correlation coefficients can illustrate the strengths of associations between two measures [45,47]. Additionally, multiple regression analyses could be used to explore the sources of differences between the two measures [4,12]. Newly available artificial intelligence (AI) machine-learning-based multivariate analytics, such as the generalized regression (GR) method, can be used to identify significant factors that could contribute to the differences between two measures with added analytic criteria to enhance prediction accuracy [4,48,49].

The trend on the common use of smartphones and with most mobile-phone users accessing Internet-based health apps, mobile apps might be used to facilitate personalized nutrition in preparation for the mobile health (mHealth) era via an accurate assessment of essential micro-nutrients [42,50,51]. The mHealth apps with data analytics platforms might also help improve access to quality nutrition data for a faster assessment of key micro-nutrients with intakes on fruits, vegetables, fats, and sugar-sweetened beverages to achieve personalized nutrients [52,53,54,55]. As mobile apps offer more versatility with lower respondent burden and faster feedback, they could be more cost-effective in facilitating self-monitoring of health behaviors to improve healthy eating habits [55,56,57,58,59]. The correlations between the mobile app and 24-hour recalls were moderate to high (0.3–0.95) for macro-nutrients, and the difference between the two varied (5 to >50% difference) for macro-nutrients [60,61,62]. Specifically, using 5–10% difference as criteria, mobile apps were not recommended as replacements for the reference method (1–2 days dietary recall) on macro-nutrients of calories, protein [60], and fat [60,61]. Compared to the controlled diets, a mobile app log significantly underestimated calories by 32%, protein by 56%, and fat by 68%, whereas 24-h dietary recalls significantly underestimated fat by 23% [62]. None of the prior studies validated mobile apps in assessing micro-nutrients for personalized nutrition. As a proof-of-concept, we added predictive modeling to examine the sources of the differences in assessing nutrients between the two measures. Thus, in this study, we integrated AI machine-learning-based analytics to validate the accuracy of a mobile app against FFQ on assessing key macro- and micro-nutrients across various modern diets.

## 2. Materials and Methods

We examined 135 modern human diets of real consumers taken by populations derived from prior studies [4,12,63]. Based on our research team’s [4,12,63] and other studies [22,30], under-reporting dietary intake is a common behavior. Validation with model diets can provide a more accurate estimate of nutrients compared to human reporting and deviating from needed intakes. While simulation with model diets may not represent actual human intake, validation of dietary intake with model diets can provide more accurate and controlled estimates of nutrient intakes [12,64]; therefore, we categorized human diets based on possible liquids and solid foods to enhance the delivery of macro- and micro-nutrients. Baseline daily recipes with variations of added calories, proteins, vegetables, fruits, or fats for all categories were included in these diets. We included four categories of modern diets as (1) liquid diets [35,39]; (2) convenient diets (canned food, high-school café diet, and fast foods) [8,33,34]; (3) ethnic diets of Western (American, Mexican, Italian, and Mediterranean) and Eastern (Japanese, Chinese, and Korean) origins [31,32], and (4) smoothies added to these diets [35,36,37,38].

We examined the macro- and micro-nutrients based on the National Institute of Health’s (NIH) dietary nutrients to meet the needs of 97–98% of healthy adults across all demographics in the US [13] with both the FFQ and the mobile app. The differences between the two measures were assessed across all identified macro- and micro-nutrients. Macro-nutrients included energy-producing carbohydrates, protein, and total fat, as well as saturated fat, cholesterol, and fiber. Micro-nutrients including B vitamins (B1 thiamin, B2 riboflavin, B3 niacin, B6 pyridoxine, B9 folate, and B12 cobalamin), vitamin C, vitamin A, vitamin D, vitamin E, choline; minerals (zinc, calcium, magnesium, iron, and sodium); essential amino acids methionine and glycine [13]. Micro-nutrients that are fat-soluble include vitamins A, D, and E; water-soluble micro-nutrients include vitamins Bs and C. Foods containing meat-based protein also have fat, saturated fat, and cholesterol with high content of vitamin B12, methionine, and glycine. Foods containing carbohydrates also have fiber with high content of vitamins A, B9, and C [13].

### 2.1. Selected Modern Human Diets

As many diets were reported with insufficient essential nutrients [8,40,41], we have categorized liquids and smoothies [35,36,37,38,39] as important modern diets in healthcare and community settings. Additionally, convenient diets [33,34] are common modern diets in addition to various ethnic diets [31,32]. The following sections present the four categories of modern human diets, including liquid diets, convenient diets, ethnic diets, and smoothie-added diets.

Liquid diets are most commonly used by frail elders or patients with gastrointestinal (GI) disorders or recovering from surgery and illness, or in palliative care settings for terminally ill cancer patients in modern healthcare settings [35,39]. Additionally, liquid diets are essential for humans to maintain basic hydration, and healthy foods are necessary to maintain GI motility and for the microbiome in the human GI system to enhance immune functions for human health [65,66]. The liquid diets evaluated in this study embodied items such as jello, ice pops, teas, coffee, Gatorade, Ensure, fruit juices, vegetable juices, and soups of various kinds, including pure liquids to cream soups, and cream of wheat commonly provided in healthcare settings.

Convenient diets provide access to various nutrients in modern industrial productive societies. The convenient diets evaluated in this study included subtypes of canned-food diets, high-school café diets, and fast-food diets [8,33,34]. The canned-food diets embodied canned soups, corn, beans and peas, potatoes, white bread, ramen noodles, frozen vegetables, and hot dogs. The high-school diets typically contained high calories food mostly associated with increased sodium and saturated fat [8], including school cafeteria items such as pizza, cheese sticks, mini cheeseburgers, corn dogs, grilled cheese sandwiches, chicken wings, and chicken tenders. The fast-food diet encompasses processed foods rich in calories, fat, sodium, and sugar [33,34]. The fast-food diets contained popular value-priced items such as chicken nuggets, fried chicken sandwiches, hamburgers, and fries along with breakfast foods of biscuits, English-muffin-based sandwiches, breakfast wraps, and hash browns [8,33,34].

The ethnic diets evaluated in this study included diets with American, Mexican, Italian, Mediterranean, Japanese, Chinese, and Korean influences [31,32]. The American diets included steaks, fried chicken, burgers, and salads. The Mexican diets included tortillas, tacos with carnitas, enchiladas, nachos with guacamole, tamales, breakfast burritos, quesadillas, tostadas, steak burritos, and chili soups. The Italian diets consisted of lasagna, alfredo, spaghetti, pizzas, calzones, bruschetta, pasta carbonara, and zucchini eggplant lasagna. The Mediterranean diets are rich in whole grains, fruits, vegetables, nuts, olive oil with limited meat, and could include baba ghanoush, falafel, hummus, Greek salads, pita bread, bulghur, shish kebab, and lamb. The Japanese diets contained sashimi, sushi, miso soup, edamame, udon, and tempura. The Chinese diets comprised sweet and sour pork, tofu, wonton, chow mein, duck, kung pao chicken, eggs, steamed buns, and rice. The Korean diets included Kimchi stew, white rice, bulgogi meats, mixed bowls or bibimbap, soups with tofu and seaweed [31,32].

Smoothie-added diet recipes are commonly used when people need added hydration or nutrients with activities such as exercise by athletes or others undergoing physically straining activities. The smoothies evaluated in this study included a variety of fruits and vegetables added to the modern base diets. Fruits comprised apples, mangoes, bananas, strawberries, peaches, grapes, apricots, blueberries, cranberries, and melons. The vegetables included broccoli, cabbage, kale, and spinach. Ethnic Chinese, Italian, and Mediterranean fruit smoothies included ginger roots, baby spinach, beet juice, Japanese matcha smoothies, and Korean multigrain smoothies [35,36,37,38].

### 2.2. Dietary Measures and Nutrient Intakes

We assessed the selected modern diets using a 144-item for 30-day with FFQ, a 12-page questionnaire with complexities of content adjustments using frequencies and portions sizes of foods [4,67]. The FFQ consisted of three sections: (1) usual food choices, (2) usual food and beverage use, and (3) summary questions. The usual food choices section contained 13 additional questions to adjust fat intakes through detailed inquiry on food preparation methods and added fats both in cooking and at the table. The usual food and beverage use section included 128 foods or food groups with the amount and frequency of foods such as cereals, bread, snacks items, meat, fish, eggs, pasta, mixed dishes, soups items, dairy products, vegetables, grains, fruits, beverages, and alcohol. In the summary questions section, three items on intakes of fruits, vegetables, and fats added to foods and used in cooking were included [67,68]. The nutrient database used to quantify the FFQ was derived from the University of Minnesota Nutrition Coordinating Center (NCC) [69].

Dietary intakes on nutrients were also analyzed for a 3-day average using a mobile app that was developed to assess daily nutrient intakes (GB HealthWatch, San Diego, CA, USA) [42,70]. The mobile app was developed by a digital health and nutritional genomics company that focused on gene–diet interactions and research tool development (https://healthwatch360.gbhealthwatch.com, accessed on 1 June 2021). In 2016, NIH funded the company to develop a mobile app for personalized genetics-based diets to record daily food consumption for dietary management and to empower users to take control of healthy eating for disease prevention. The app had the capacity to extract 30 essential nutrients by analyzing users’ food logs and provided reports on the total amounts and percentages of suggested daily values of nutrients. Furthermore, it offered personalized recommendations based on dietary guidelines and users’ goals to manage weight and to prevent chronic conditions such as metabolic syndrome and Alzheimer’s disease [42,70]. Thus, for each diet, we performed a nutrient analysis using both 30-day FFQ and mobile app by mathematically dividing the 30-day data into a 3-day dietary diary. For example, 1 oz (30 g) of fat intake every week would yield 13 g for 3 days or 4.3 g of fat for 1 day. This calculation is comparable dietary intakes between the two measures, while realistically, many human subjects might neglect to report the minute dietary intakes; therefore, the 3-day dietary diary in this study could be more detailed than human reporting. Before analysis, data entry was checked independently by two research team members for accuracy.

### 2.3. Data Analysis

All data were analyzed using JMP version 13.0.0 statistical software [64,71,72] (SAS Institute Inc., Cary, NC, USA). We first assessed bias and agreement, and then GR to predict the source of differences between the two measures. Means and standard deviations (SD) for all nutrients [73] were calculated for both mobile App and FFQ. Then, agreement and bias analyses using mean % differences and standard errors (SE) of the differences between the two measures were compared across all nutrient parameters. Pearson correlation coefficients (*r*) were used to measure the strengths of associations between the two dietary measures (*r* > 0.80 as very strong, *r* < 0.60 as moderate) [43]. The Bland–Altman plots were used to visualize the mean % differences with the limits of agreement (LoA: mean difference ± 2 SD) between the two measures [28,72] for variability of the differences, with a good agreement if 95% and greater of the agreement being within ± 2 SDs [20,21,64]. The alpha for all analyses was set at 0.05 for the significance level.

We then utilized GR models to predict the differences between the app and FFQ in assessing essential nutrients by progressively including related factors in the dataset. The analytics and rationales have been reported earlier [4,12,63] and are summarized in the following. We added predictive modeling to examine the sources of the differences between the two measures [63]. JMP software provided default logistic regression (LR) as a baseline and exploratory model to predict dependent variables in categorical values. Following LR, other models could be selected for validation (Elastic Net) and associated validation methods (Leave-One-Out, Validation Column) for further analysis. In effect, GR estimation methods are a confirmatory model to predict the accuracy with a lower misclassification rate for minimal prediction error [4,12,64,74]. We incorporated Elastic Net models for their capacity to handle datasets with many variables, balancing potential interactions from various domain factors [75]. It is important to point out that GR eliminates certain predictors to avoid over-fitting. Conventional statistical procedures are limited by the sample size [63]. If the number of parameters to be estimated exceeds the degrees of freedom, the regression model would be highly unstable. The AI-based analytics use partition in iteration by resampling with machine learning [63]. Both AICc validation and LOO cross-validation methods are effective methods for small sample sizes and handling multiple domains based on the logic of resampling [4,72]. In resampling, observed biases are corrected by such repeated analyses on random subsets [63]. This AI machine learning approach is superior to conventional statistics, including the baseline logistic regression analyses that tend to yield an overfitted model [4,63]. We used Leave-One-Out (LOO) for validations to select significant factors within domains of caloric ranges, the effect of differences from energy nutrients on the differences on micro-nutrients, and diet types [48,49]. Once significant factors were determined through LOO within each domain, we then used AICc validation columns to confirm how well the model fits with unbiased prediction [49].

We identified related factors that could affect the differences between FFQ and the app per categories of (1) caloric ranges (<1000, 1000–2000, or >2000) of total calories; (2) effects of differences from energy-producing carbohydrate, protein, and fat; (3) diet types. We used an 80/20 split for training and validation sets for predictive modeling to further identify significant predictors that contributed to the difference between the app and FFQ. The best models on predicting the sources contributing to the differences were based on three accuracy criteria, including lowest AICc (fitter for more precise model), lower misclassification (smaller for accuracy), and higher area under the receiver operating characteristics (ROC) curve (AUC, >0.80) [12,64]. The prediction and interaction profilers were used to visualize the potential significant interactions among the factors. If significant interactions exist in the models, the interaction terms would be included in the model testing [48,49].

## 3. Results

### 3.1. Agreement and Difference: The Bland and Altman Method

We examined the data using the Bland and Altman method to assess the agreement (% differences), bias (SE), and correlations between FFQ and mobile app per three caloric ranges (<1000, 1000–2000, and >2000) and all assessed nutrients (Table 1) using 5–10% differences as criteria [60]. Compared to FFQ, the mobile app presented acceptable differences (<5%) with a caloric range of <1000 but underestimated the caloric range of 1000–2000 calories (>5%) and >2000 (>10%). For macro-nutrients, the app underestimated total calories and protein (>5%); fat, saturated fat, and cholesterol (>10%); overestimated fiber (>10%); presented acceptable differences with carbohydrate (<5%). For micro-nutrients, the app underestimated choline, vitamin D (>5%); vitamin B12, methionine, glycine, vitamin E, zinc, and sodium (>10%); overestimated vitamins A and C (>5%); presented acceptable differences with other B vitamins including B1, B2, B3, B6, B9, and calcium, magnesium, and iron. Thus, we found good agreement between the two measures on many micro-nutrients but not protein-based micro-nutrients that are essential in nutrigenomics pathways.

The bias (SE) increased between the two measures with increased caloric ranges was smallest for 1000–2000 caloric range (1.44), larger for <1000 (2.3), and largest for >2000 (5.91); indicating the greater spread of means. The bias was greater (>2) for fiber, vitamins A and C, calcium, and sodium when compared to all other nutrients. The correlations between the two measures were strong for the mid-range of 1000–2000 caloric range (0.78), moderate for higher calories of >2000 (0.64), and lowest for <1000 calories intake (0.54) (average 0.65) (all *p* < 0.001). The correlations are strong for most nutrients (average for macro- 0.87 and 0.84 for micro-nutrients) except for calcium (0.53).

**Figure 1:**
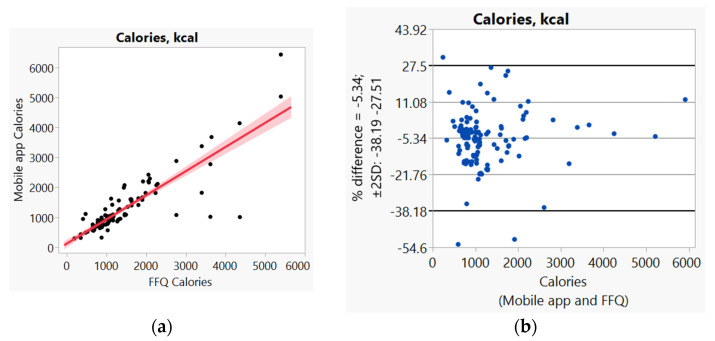
(**a**) Correlation, (**b**) Bland and Altman plots between mobile application and Food Frequency Questionnaire (FFQ) on total calories.

**Figure 2:**
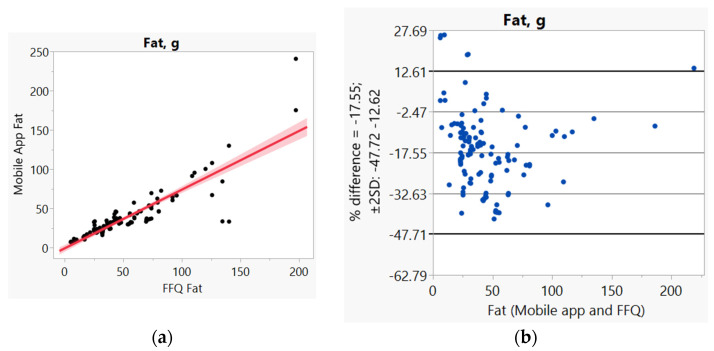
(**a**) Correlation, (**b**) Bland and Altman plots between mobile application and Food Frequency Questionnaire (FFQ) for fat.

**Figure 3:**
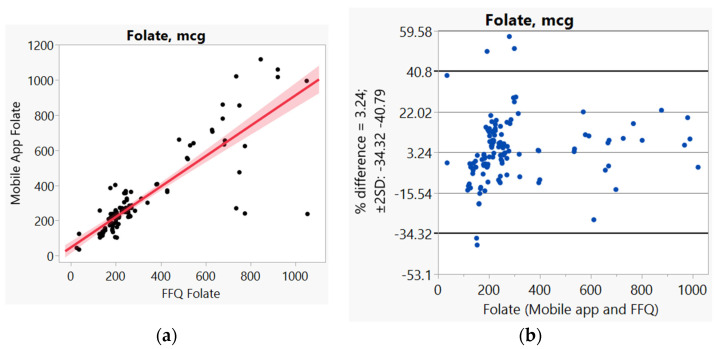
(**a**) Correlation, (**b**) Bland and Altman plots between mobile application and Food Frequency Questionnaire (FFQ) for folate.

**Figure 4:**
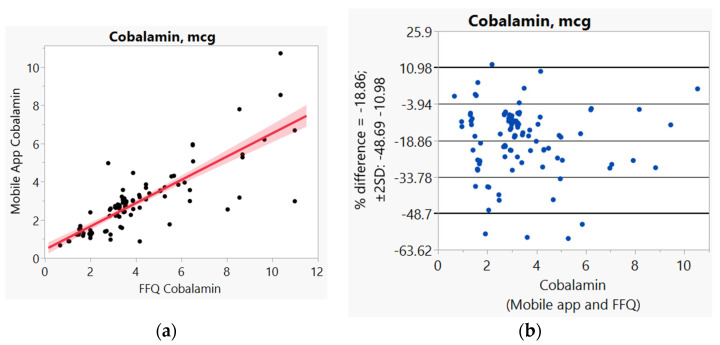
(**a**) Correlation, (**b**) Bland and Altman plots between mobile applications and Food Frequency Questionnaire (FFQ) for cobalamin.

In addition, we used Bland–Altman plots to visualize the difference and LoA between mobile app and FFQ. We presented side-by-side comparison of correlation (left panel) and Bland–Altman plots (right panel) on example macro-nutrients of calories (Figure 1a,b), fat (Figure 2a,b), protein (Figure S1a,b), carbohydrate (Figure S2a,b); and micro-nutrients of folate (Figure 3a,b) and cobalamin (Figure 4a,b). Correlation plots presented good agreement between the two measures for all nutrients presented in these figures. Bland–Altman plots displayed a wide variability of differences for both macro- (calories, fat, protein, carbohydrate) and micro-nutrients (folate, cobalamin). Bland–Altman plots further illustrated the underestimation by the app compared to FFQ on total calories (−5.34%, Figure 1b), fat (−17.55%, Figure 2b), protein −9.8%, Figure S1b), and cobalamin (−18.86%, Figure 4b); but higher estimation on carbohydrate (4.55%, Figure S2b) and folate intakes (3.2%, Figure 3b).

Furthermore, we assessed the differences between the mobile app and FFQ with nutrients in association with caloric ranges and diets (Table 2). For total calories with caloric ranges, the app, in comparison to FFQ, presented acceptable differences with <1000 calories (<5%); but underestimated with 1000–2000 calories (>5%) and >2000 calories (>10%) (Table 2). Additionally, the app overestimated total calories with pure liquid diet (>10%), underestimated total calories with high school, Mediterranean, Chinese, and smoothie-added diets (>5%); also canned food and fast-food diets with greater bias (>10%), but presented acceptable differences (<5%) with FFQ on American, Mexican, Italian, Japanese, and Korean diets. Likewise, the app overestimated carbohydrates with a caloric range of <2000, but underestimated >2000 caloric range (>5%); with Italian, Japanese, Korean diets (>5%); pure liquid, Mexican, Chinese diets (>10%); but presented acceptable differences (<5%) with other diets (canned food, high school, fast food, American, Mediterranean, and smoothie-added). Additionally, the app underestimated protein with caloric ranges of 1000–2000 (>5%), and caloric ranges of <1000 and >2000 (>10%); pure liquid, high school, fast food, American, Mexican, Italian, Japanese, Chinese diets (>5%); canned food, Mediterranean, Korean, and smoothie-added diets (>10%). The app underestimated fat with all caloric ranges (>10%) and all diets except liquid diets. For folate, the app presented overestimation with a caloric range of 1000–2000, underestimation with a caloric range of >2000, and acceptable differences (<5%) with a caloric range of <1000; underestimation with Mediterranean diets; overestimation with American and Italian diets (>5%); pure liquid, Mexican, and Chinese diets (>10%); but acceptable differences (<5%) with canned food, high school, fast food, Japanese, Korean, and smoothie-added diets. For cobalamin, the app presented underestimation with all caloric ranges (>10%) and all diets except fast-food diets. Additionally, the app underestimated protein-based and fat-based nutrients, including saturated fat, cholesterol, methionine, choline, glycine, vitamins B12 and E; but overestimated carbohydrate-based nutrients including fiber, vitamins B9, A, and C (Tables S1–S4).

### 3.2. Predictive Modeling for the Difference of Mobile App against FFQ: Generalized Regression Analysis

**Figure 5:**
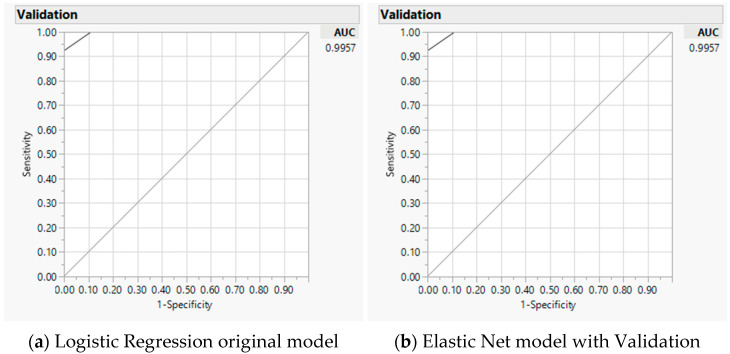
Predicting accuracy of total calories analyses using mobile application against Food Frequency Questionnaire: Area under the receiver operating characteristic curve (AUC) for baseline logistic regression model (**a**) and Elastic Net with validation model (**b**).

For predictive modeling, we progressively examined significant factors per individual domains of caloric ranges (coded as one of the three versus the other two categories for <1000, 1000–2000, and >2000), energy nutrients, and various diets. We included the significant factors of all domain factors in the final combined model (Table S5 progression examples for total calories, Table S6 for folate, Table S7 for cobalamin). For total calories, differences on 1000–2000 over other caloric ranges, carbohydrate, and protein were significant contributing factors to the difference between two measures (misclassification 0.04, AICc 18.2, and AUC 0.99) (Table 3), baseline LR model on the left panel and GR model validation on the right panel. As an example, Figure 5 illustrates the AUC curve with closer to 100% sensitivity and 100% specificity for the accuracy of the selected model with total calories [48]. Through the progression analyses, we noted a higher AICc and less precise model by including the additional factor of the Japanese diet, thus a less favorable model than the selected model (Table S5).

Folate and cobalamin are the most representative essential micro-nutrients needed in the nutrigenomics pathways. Factors that contributed to the differences in folate between the two measures included caloric range (1000–2000 versus other two categories), carbohydrate, fiber, and Mediterranean diet (misclassification 0.11, AICc 30.7, and AUC 0.91) (Table 4). Through the progression analyses, we noted a higher AICc and lower AUC, thus, less precise and less favorable models than the selected model by including additional factors of fiber and Chinese diet (Table S6).

For cobalamin, significant factors that contributed to the differences between the two measures included caloric range (1000–2000 versus other two categories), protein, and Chinese diet (misclassification 0.27, AICc 35.8, and AUC 0.79) (Table 5). With the progression analyses, we observed a higher AICc by including additional factors of fat and Korean diet, which presented less favorable models than the selected model (Table S7).

We also examined significant factors between the two measures for other nutrients with the progression analyses and summarized final models for other nutrients (carbohydrate, protein, fat, saturated fat, cholesterol, and fiber in Table S8; thiamin, riboflavin, niacin, pyridoxine, choline, glycine, and zinc in Table S9; and vitamins A, C, D, E, calcium, magnesium, iron, sodium in Table S10). Significant factors that contributed to the difference between the two measures on carbohydrate included calories, fat, fiber, and Mediterranean diet; for protein: caloric range (1000–2000 versus others), calories, and fat; on fat: calories, saturated fat, and Japanese diet; on saturated fat: fat, cholesterol, and Japanese diet; for cholesterol: protein, saturated fat, and Japanese diet; on fiber: carbohydrate, Japanese diet, and Chinese diet (misclassification 0.04–0.23, AICc 20.4–33.8, AUC 0.74–0.98). Similarly, significant factors for thiamin included caloric range (<1000 versus others), saturated fat, fiber, and canned-food diet; for riboflavin: protein, fiber, and canned-food diet; for niacin: calories, fiber, canned food, and Italian diet; for pyridoxine: calories, fiber, and Japanese diet; for choline: calories, protein, and canned-food diet; for glycine: protein, Mexican, and Japanese diet; and for zinc: protein, canned food, fast-food, and Japanese diet (misclassification 0.04–0.29, AICc 20.5–36.53, AUC 0.82–0.96). Additionally, significant factors for vitamin A included fat, saturated fat, and fast-food diet; vitamin C: caloric range (<1000 versus others), fiber, and canned-food diet; on vitamin D: protein, fat, canned food, and Chinese diet; for vitamin E; carbohydrate, fat, and cholesterol; for calcium; carbohydrate, cholesterol, American, and Italian diet; on magnesium: carbohydrate, fat, cholesterol, and fiber; for iron: calories, protein, fiber, and Italian diet; on sodium: protein, fiber, and high school diet (misclassification 0.18–0.36, AICc 29.6–39.9, AUC 0.75–0.96). For diet types, the Japanese diet was a common contributing factor for the differences between the two measures on fat, saturated fat, cholesterol, fiber, riboflavin, pyridoxine, glycine, and zinc (Tables S8–S10). We did not explicitly test the model for methionine, as methionine was purely dependent on protein. The interaction profiler plots did not present any significant three-way interactions in the final models for all nutrients.

## 4. Discussion

In preparation for personalized nutrition, we validated the accuracy of a mobile app against the FFQ as a reference method in assessing key nutrients with various modern diets. Using 5–10% difference as criteria [60], compared to FFQ, the app presented acceptable estimation with a caloric range of <1000 but not for total calories and caloric ranges of 1000 or higher with a greater bias for calories of >2000. Specifically, for macro-nutrients, the app presented acceptable estimation for carbohydrates but underestimated protein and greatly underestimated fats (fat, saturated fat, cholesterol). For micro-nutrients, the app presented with acceptable estimation for most B vitamins (B1, B2, B3, B6, and B9) and some minerals (calcium, magnesium, and iron), but underestimated choline and vitamin D and greatly underestimated vitamin B12, methionine, glycine, vitamin E, zinc, and sodium; and greatly overestimated vitamins A and C. Prior research indicated that mobile apps were not recommended as replacements to reference method (1–2 day dietary recall) on macro-nutrients of calories, protein [60], and fat [60,61]. With this study, we further demonstrated that the mobile app presented with greater bias with increased calories with FFQ as a reference method, while biases are similar with total calories, protein, and fat. Although correlations between the two measures were strong, lower correlations were observed with greater calories of the diets and calcium.

Furthermore, we demonstrated that caloric ranges and various diets may be used to examine additional sources that might have contributed to the differences between the two measures. Overall, the app underestimated all macro- and micro-nutrients (except vitamins B1 and A) with calories >2000, underestimated protein-based and fat-based nutrients, but overestimated carbohydrate-based nutrients for calories of ≤2000. Additionally, we noted that with various diets, the app underestimated foods that contain meat-based protein and fat (saturated fat, cholesterol, vitamins B12 and E, methionine, choline, glycine), but overestimated foods that contain carbohydrate nutrients (fiber, vitamins B9, A, and C). We used FFQ as the reference method for its capacity to adjust fat intakes [22,24,25,26]. Hence, additional adjustments with caloric ranges and diet types could be considered for the mobile app. Previous studies used <500 and >3500 calories as the limits for valid caloric ranges of FFQ that were extreme for human health [4,12,22,30]. With the realistic caloric intakes for human health, the challenges of accuracy remain for the mobile app.

As a proof-of-concept, we added predictive modeling to examine the sources that contributed to the differences between the two measures [4,63,64]. We used AI machine-learning-based analytics with criteria to enhance the accuracy of prediction. Thus far, no prior studies have validated the accuracy of a mobile app against FFQ using predictive modeling. With confirmatory predictive modeling, we demonstrated that caloric ranges and source of micro-nutrients in relation to macro-nutrients (folate source of difference from carbohydrates versus cobalamin source of difference from protein) contributed to less accurate estimates of nutrients for the mobile app in reference to FFQ. A specific diet, such as Mediterranean (folate) and Chinese (cobalamin) diets, might further contribute to the differences between the two measures; therefore, caloric ranges and source of macro-nutrients might be considered for adjustments to accurately measure micro-nutrients.

In summary, we noted that fat and protein were major sources for the differences between FFQ and mobile app, with specific caloric ranges and diet types contributing to the differences. The predictive modeling further substantiated the findings that fat and protein were the major sources of differences between the two measures, with specific caloric ranges and diet types contributing to the differences. The challenges remain for the mobile app to accurately measure macro- and micro-nutrients. Further adjustments with caloric ranges, source of nutrients, and diets might help to improve the accuracy of the mobile app. In addition, future studies may include various diets across different human populations with accurate measures on dietary intakes and nutrients with the mobile app. In conclusion, the mobile app has the capability to support personalized nutrition in the mHealth era with the use of AI-based analytics, integrating potential contributing factors to improve its accuracy.

## Figures and Tables

**Table 1:** Agreement and bias for the mobile application against Food Frequency Questionnaire (*N* = 135).

Parameters	% Difference M ± SD	FFQ M ± SD	Mobile M ± SD	SE	± 2 SD%	*r* **
Calories (kcal)	−5.34 ** ± 16.42	1332 ± 904.4	1201 ± 8464	1.41	94.81	0.86
<1000 (*N* = 54)	−0.88 ± 16.87	748.3 ± 170.4	730.3 ± 182.1	2.30	94.44	0.54
1000–2000 (*N* = 63)	−7.08 ** ± 11.46	1297 ± 290.4	1180 ± 365.4	1.44	94.33	0.78
>2000 (*N* = 18)	−12.63 * ± 25.09	3206 ± 1114	2685 ± 1421	5.91	88.89	0.64
Carbohydrate (g)	4.55 ** ± 19.58	169.4 ± 132.0	176.8 ± 134.7	1.68	91.85	0.85
Protein (g)	−9.80 ** ± 12.27	54.84 ± 36.21	46.71 ± 32.88	1.06	94.81	0.87
Fat (g)	−17.55 ** ± 15.08	50.80 ± 34.29	37.20 ± 29.35	1.30	94.07	0.87
Sat Fat (g)	−17.69 ** ± 15.99	15.32 ± 10.72	11.37 ± 9.28	1.38	95.56	0.88
Cholesterol (mg)	−10.26 ** ± 13.37	197.8 ± 131.2	172.4 ± 121.6	1.15	95.56	0.91
Fiber (g)	11.39 ** ± 24.03	15.97 ± 16.65	18.19 ± 18.73	2.07	93.33	0.85
Thiamin (mg)	2.46 ± 15.59	1.08 ± 0.70	1.11 ± 0.72	1.34	94.81	0.86
Riboflavin (mg)	−1.75 ± 15.11	1.27 ± 0.83	1.23 ± 0.80	1.30	93.33	0.86
Niacin (mg)	−0.91 ± 16.35	14.63 ± 9.93	14.16 ± 10.30	1.41	91.85	0.87
Pyridoxine (mg)	3.29 * ± 19.29	1.52 ± 1.29	1.58 ± 1.40	1.66	93.33	0.84
Folate (mcg)	3.24 * ± 18.78	290.7 ± 209.4	298.9 ± 214.2	1.62	93.33	0.85
Cobalamin (mcg)	−18.86 ** ± 14.92	3.71 ± 2.10	2.69 ± 1.54	1.28	94.07	0.83
Methionine (g)	−11.90 ** ± 12.93	1.23 ± 0.81	1.01 ± 0.74	1.11	92.59	0.88
Choline (mg)	−6.23 ** ± 15.42	266.4 ± 176.4	237.7 ± 160.4	1.33	94.07	0.86
Glycine (g)	−12.13 ** ± 14.55	2.31 ± 1.58	1.89 ± 1.44	1.25	92.59	0.87
Vitamin A (IU)	35.19 ** ± 29.40	11,037 ± 14,343	16,238 ± 16,359	2.53	97.04	0.87
Vitamin C (mcg)	18.27 ** ± 30.07	123.4 ± 137.5	148.2 ± 165.8	2.59	92.59	0.86
Vitamin D (mcg)	−6.18 ** ± 12.81	4.38 ± 2.42	3.95 ± 2.19	1.10	93.33	0.90
Vitamin E (mcg)	−32.85 ** ± 18.95	10.87 ± 7.42	6.09 ± 4.84	1.63	91.85	0.83
Zinc (mg)	−10.44 ** ± 15.38	7.39 ± 4.62	6.15 ± 3.87	1.32	93.33	0.86
Calcium (mg)	−2.66 ± 31.33	594.4 ± 382.8	569.3 ± 383.8	2.70	93.33	0.53
Magnesium (mg)	3.72 * ± 17.26	201.1 ± 150.3	208.8 ± 157.6	1.49	93.33	0.85
Iron (mg)	1.46 ± 17.42	9.00 ± 5.81	8.91 ± 5.60	1.50	93.33	0.88
Sodium (mg)	−12.60 ** ± 24.94	2551 ± 1700	1969 ± 1263	2.15	97.04	0.83

Note: M: mean; SD: standard deviation; FFQ: food frequency questionnaire; SE: standard error; * p < 0.05; ** p < 0.001.

**Table 2:** Differences between the mobile application and Food Frequency Questionnaire per domains of caloric ranges and various diets for key macro- and micro-nutrients (*N* = 135).

Parameters (*N*)	Calories, kcal	Carbohydrate, g	Protein, g	Fat, g	Folate, mcg	Cobalamin, mcg
	%diff M ± SD	%diff M ± SD	%diff M ± SD	%diff M ± SD	%diff M ± SD	%diff M ± SD
Caloric ranges						
<1000 (54)	−0.88 ± 16.87	7.40 ** ± 19.79	−10.77 ** ± 10.43	−11.75 ** ± 13.66	2.06 ± 20.12	−21.02 ** ± 16.02
1000–2000 (63)	−7.08 ** ± 11.46	5.91 ** ± 14.34	−6.93 ** ± 9.31	−22.60 ** ± 11.58	7.04 ** ± 12.56	−14.87 ** ± 12.47
>2000 (18)	−12.63 * ± 25.09	−8.78 ± 28.79	−16.93 ** ± 21.09	−17.28 ** ± 22.83	−6.53 ± 27.99	−26.31 ** ± 15.94
Diet Types						
Pure liquid (10)	16.28 ± 26.70	23.27 * ± 29.82	−9.14 ± 12.82	−3.23 ± 18.02	20.44 ± 35.87	−20.13 * ± 25.65
Convenient Diet (30)	−12.39 ** ± 9.61	−0.34 ± 10.15	−9.63 ** ± 11.74	−26.68 ** ± 10.20	0.76 ± 13.84	−9.82 ** ± 10.84
Canned Food (10)	−12.98 ** ± 5.62	−3.99 * ± 5.39	−12.68 ** ± 7.27	−27.05 ** ± 5.42	−3.11 ± 14.39	−9.95 ** ± 1.72
High School (10)	−8.33 ** ± 2.99	2.24 ± 3.46	−8.40 ** ± 4.78	−18.58 ** ± 5.20	4.51 ± 8.19	−15.43 ** ± 6.48
Fast Food (10)	−15.86 ** ± 15.01	0.72 ± 16.35	−7.80 ± 18.79	−34.40 ** ± 11.82	0.87 ± 17.63	−4.07 ± 16.18
Ethnic Food (73)	−5.00 ** ± 9.94	6.21 ** ± 15.43	−9.27 ** ± 8.46	−17.50 ** ± 9.68	4.11 ** ± 12.80	−19.96 ** ± 11.24
Western Diet (40)	−4.49 ** ± 9.77	5.20 ± 16.46	−8.47 ** ± 8.00	−14.25 ** ± 8.38	3.64 ± 13.74	−16.46 ** ± 11.64
American (10)	−4.16 ± 14.42	3.87 ± 22.58	−5.14 ± 7.60	−15.16 ** ± 6.97	5.43 ± 17.35	−9.73 ** ± 6.53
Mexican (10)	−4.90 ± 10.69	10.81 ± 20.60	−6.99 ** ± 4.79	−22.07 ** ± 3.96	11.42 * ± 13.22	−12.92 ** ± 4.78
Italian (10)	−3.43 ** ± 3.18	6.61 ** ± 4.69	−6.10 * ± 6.86	−14.07 ** ± 3.27	7.29 ** ± 1.77	−15.71 ** ± 9.93
Mediterranean (10)	−5.45 ± 8.88	−0.48 ± 11.92	−15.66 ** ± 8.43	−5.69 ± 8.87	−9.60 ** ± 7.61	−27.48 ** ± 14.94
Eastern Diet (33)	−5.63 ** ± 10.26	7.44 ** ± 14.24	−10.24 ** ± 9.01	−21.45 ** ± 9.80	4.68 * ± 11.74	−24.20 ** ± 9.24
Japanese (10)	−3.02 ** ± 1.29	5.05 ** ± 2.17	−5.08 ** ± 0.77	−12.29 ** ± 1.19	−2.25 ** ± 1.14	−16.76 ** ± 0.43
Chinese (10)	−9.74 ** ± 5.33	12.34 ** ± 7.03	−9.44 ** ± 1.85	−32.50 ** ± 3.75	15.64 ** ± 2.62	−20.75 ** ± 0.84
Korean (13)	−4.47 ± 15.39	5.52 ± 21.72	−14.82 ** ± 12.96	−20.00 ** ± 8.39	1.57 ± 14.52	−32.58 ** ± 9.74
Smoothie-added (22)	−6.67 ± 25.56	−2.83 ± 28.95	−12.09 * ± 21.17	−14.70 ** ± 22.68	−4.09 ± 25.77	−26.94 ** ± 18.78

Note: M: mean; SD: standard deviation; * p < 0.05; ** p < 0.001.

**Table 3:** Significant factors contributing to the differences between the mobile application and Food Frequency Questionnaire on total calories.

Parameters	Logistic Regression Original Model		Generalized Regression Elastic Net Model Validation	
	Estimate	*p* (*χ*^2^)	Estimate	*p* (*χ*^2^)
(Intercept)	1.66	0.0160	1.66	0.0035
1000–2000 caloric range	−2.79	0.0003	−2.79	<0.0001
Carbohydrate % Difference	3.24	<0.0001	3.24	<0.0001
Protein % Difference	−3.05	<0.0001	−3.05	<0.0001
MR	0.0455		0.0455	
AICc	18.18		18.19	
AUC	0.9957		0.9957	

Note: MR: Misclassification rate; AICc: Akaike’s information criterion with corrections; AUC: Area under the curve.

**Table 4:** Significant factors contributing to the differences between mobile application a nd Food Frequency Questionnaire on folate.

Parameters	Logistic Regression Original Model		Generalized Regression Elastic Net Model Validation	
	Estimate	*p* (*χ*^2^)	Estimate	*p* (*χ*^2^)
(Intercept)	−5.59	0.9386	−3.83	<0.0001
1000–2000 caloric range	−1.81	0.0053	−1.81	0.0084
Carbohydrate % Difference	−2.35	0.0006	−2.35	0.0003
Fiber % Difference	−2.07	0.0008	−2.07	0.0008
Mediterranean	9.11	0.9001	7.35	<0.0001
MR	0.1154		0.1154	
AICc	30.71		30.73	
AUC	0.9125		0.9125	

Note: MR: Misclassification rate; AICc: Akaike’s information criterion with corrections; AUC: Area under the curve.

**Table 5:** Significant factors contributing to the differences between mobile application and Food Frequency Questionnaire on cobalamin.

Parameters	Logistic Regression Original Model		Generalized Regression Elastic Net Model Validation	
	Estimate	*p* (*χ*^2^)	Estimate	*p* (*χ*^2^)
(Intercept)	11.63	0.8783	3.87	<0.0001
1000–2000 caloric range	2.06	<0.0001	1.86	<0.0001
Protein % Difference	−1.22	0.0094	−1.05	0.0140
Chinese	−12.26	0.8718	−4.45	<0.0001
MR	0.2727		0.2727	
AICc	35.84		35.78	
AUC	0.7906		0.7906	

Note: MR: Misclassification rate; AICc: Akaike’s information criterion with corrections; AUC: Area under the curve.

## Supplementary Materials

The following are available online at https://www.mdpi.com/article/10.3390/nu14030537/s1, Table S1: Bias and agreement between Mobile Application and Food Frequency Questionnaire per domains of caloric ranges, energy nutrients, and various diets for major nutrients (*N* = 135). Table S2: Bias and agreement between mobile application and Food Frequency Questionnaire per domains of caloric ranges, energy nutrients, and various diets for vitamins Bs (*N* = 135). Table S3: Bias and agreement between mobile application and Food Frequency Questionnaire per domains of caloric ranges, energy nutrients, and various diets for methyl-donors and vitamins A, D, and D (*N* = 135). Table S4: Bias and agreement between mobile application and Food Frequency Questionnaire per domains of caloric ranges, energy nutrients, and various diets for vitamin E and minerals (*N* = 135). Table S5: Progression on selecting significant factors contributing to the differences between mobile application and Food Frequency Questionnaire on total calories. Table S6: Progression on selecting significant factors contributing to the differences between mobile application and Food Frequency Questionnaire on folate. Table S7: Progression on selecting significant factors contributing to the differences between mobile application and Food Frequency Questionnaire on cobalamin. Table S8: Summary on significant factors contributing to the differences between mobile application and Food Frequency Questionnaire on major nutrients. Table S9: Summary on significant factors contributing to the differences between mobile application and Food Frequency Questionnaire on methyl-donors and co-factors. Table S10: Summary on significant factors contributing to the differences between mobile application and Food Frequency Questionnaire on other vitamins and minerals. Figure S1: (a) Correlation, (b) Bland and Altman plots between mobile application and Food Frequency Questionnaire for protein. Figure S2: (a) Correlation, (b) Bland and Altman plots between mobile application and Food Frequency Questionnaire for carbohydrates.
