# Supplementary material for: Validating Accuracy of a Mobile Application against Food Frequency Questionnaire on Key Nutrients with Modern Diets for mHealth Era
Source: Nutrients. 2022 Jan 26;14(3):537. doi: 10.3390/nu14030537 (PMC8839756; doi:10.3390/nu14030537)
Supplement: Supplementary file 1 [file nutrients-14-00537-s001.zip › nutrients-1497124-supplementary materials.pdf]

Supplementary Table S1. Bias and agreement between Mobile Application and Food Frequency Questionnaire per domains of caloric ranges, energy nutrients, and various diets for major nutrients (*N*=135).

**Parameters (*N*)**	**Calories, kcal**<br>%diff M ± SD<br>*r***	**Carb, g**<br>%diff M ± SD<br>*r***	**Protein, g**<br>%diff M ± SD<br>*r***	**Fat, g**<br>%diff M ± SD<br>*r***	**Sat Fat, g**<br>%diff M ± SD<br>*r***	**Cholesterol, mg**<br>%diff M ± SD<br>*r***	**Fiber, g**<br>%diff M ± SD<br>*r***
Calories							
<1000 (54)	−0.88 ± 16.87<br>0.54	7.40** ± 19.79<br>0.58	−10.77** ± 10.43<br>0.80	−11.75** ± 13.66<br>0.86	−12.73** ± 15.39<br>0.71	−9.19** ± 13.26<br>0.97	14.06** ± 24.59<br>0.80
1000-2000 (63)	−7.08** ± 11.46<br>0.78	5.91** ± 14.34<br>0.91	−6.93** ± 9.31<br>0.91	−22.60** ± 11.58<br>0.81	−22.47** ± 13.27<br>0.82	−10.06** ± 10.70<br>0.94	14.25** ± 16.37<br>0.98
>2000 (18)	−12.63* ± 25.09<br>0.64	−8.78 ± 28.79<br>0.56	−16.93** ± 21.09<br>0.70	−17.28** ± 22.83<br>0.76	−15.80** ± 21.64<br>0.76	−14.19** ± 20.59<br>0.75	−6.65 ± 35.96<br>0.48
Energy Nutrients							
Fat	−26.01** ± 12.41<br>0.76	−12.94** ± 15.03<br>0.37	−24.50** ± 14.53<br>0.49	-	−27.59** ± 11.29<br>0.90	-24.86** ± 15.51<br>0.36	-15.51** ± 14.77<br>0.39
Protein	−15.16** ± 11.87<br>0.89	−5.39** ± 10.32<br>0.25	-	−0.61 ± 21.37<br>0.71	−13.69** ± 12.92<br>0.72	-14.56** ± 12.78<br>0.62	-6.78** ± 10.45<br>0.18
Carbohydrate	−5.49* ± 18.10<br>0.92	-	−3.57 ± 19.43<br>0.79	−14.46** ± 12.36<br>0.60	−0.25 ± 21.46<br>0.54	1.75 ± 23.72<br>0.51	14.15** ± 16.22<br>0.65
Diet type							
Pure Liquid (10)	16.28 ± 26.70<br>0.56	23.27* ± 29.82<br>0.56	−9.14 ± 12.82<br>0.87	−3.23 ± 18.02<br>0.83	−4.14 ± 19.19<br>0.69	−22.29** ± 20.20<br>0.45	31.98* ± 44.09<br>0.94
Convenient Diet (30)	−12.39** ± 9.61<br>0.91	−0.34 ± 10.15<br>0.85	−9.63** ± 11.74<br>0.94	−26.68** ± 10.20<br>0.91	−25.57** ± 13.63<br>0.91	−14.84** ± 13.22<br>0.97	11.38** ± 14.93<br>0.79
Canned Food (10)	−12.98** ± 5.62<br>0.76	−3.99* ± 5.39<br>0.80	−12.68** ± 7.27<br>0.98	−27.05** ± 5.42<br>0.92	−31.43** ± 3.27<br>0.82	−27.09** ± 2.50<br>0.82	20.41** ± 15.79<br>0.97
High School (10)	−8.33** ± 2.99<br>0.95	2.24 ± 3.46<br>0.96	−8.40** ± 4.78<br>0.95	−18.58** ± 5.20<br>0.91	−11.47** ± 8.35<br>0.83	−6.99** ± 5.56<br>0.86	9.13** ± 6.32<br>0.88
Fast Food (10)	−15.86** ± 15.01<br>-0.35	0.72 ± 16.35<br>0.06	−7.80 ± 18.79<br>-0.26	−34.40** ± 11.82<br>−0.18	−33.81** ± 13.54<br>−0.03	−10.44 ± 16.41<br>0.71	4.59 ± 16.78<br>-0.60
Ethnic Food (73)	−5.00** ± 9.94<br>0.94	6.21** ± 15.43<br>0.90	−9.27** ± 8.46<br>0.95	−17.50** ± 9.68<br>0.92	−17.25** ± 12.74<br>0.91	−5.89** ± 6.52<br>0.96	12.35** ± 17.01<br>0.93
Western Diet (40)	−4.49** ± 9.77<br>0.84	5.20 ± 16.46<br>0.67	−8.47** ± 8.00<br>0.83	−14.25** ± 8.38<br>0.94	−11.76** ± 12.05<br>0.89	−4.64** ± 6.12<br>0.90	9.09** ± 18.17<br>0.75
American (10)	−4.16 ± 14.42<br>0.20	3.87 ± 22.58<br>0.47	−5.14 ± 7.60<br>0.49	−15.16** ± 6.97<br>−0.07	−13.16** ± 8.29<br>-0.15	−0.86 ± 4.26<br>0.74	11.02 ± 28.90<br>0.82
Mexican (10)	−4.90 ± 10.69<br>0.69	10.81 ± 20.60<br>0.54	−6.99** ± 4.79<br>0.83	−22.07** ± 3.96<br>0.84	−21.10** ± 4.78<br>0.79	−9.12** ± 1.71<br>0.30	16.54** ± 13.29<br>0.58
Italian (10)	−3.43** ± 3.18<br>0.56	6.61** ± 4.69<br>0.66	−6.10* ± 6.86<br>-0.02	−14.07** ± 3.27<br>0.38	−13.12** ± 4.50<br>0.09	−2.75 ± 5.59<br>0.03	8.67** ± 8.36<br>0.74
Mediterranean (10)	−5.45 ± 8.88<br>0.04	−0.48 ± 11.92<br>0.40	−15.66** ± 8.43<br>0.52	−5.69 ± 8.87<br>0.22	0.32 ± 15.94<br>−0.02	−5.81 ± 8.15<br>0.65	0.13 ± 13.92<br>0.60
Eastern Diet (33)	−5.63** ± 10.26<br>0.97	7.44** ± 14.24<br>0.97	−10.24** ± 9.01<br>0.98	−21.45** ± 9.80<br>0.92	−23.89** ± 10.26<br>0.95	−7.41** ± 6.76<br>0.98	16.31** ± 14.81<br>0.97
Japanese (10)	−3.02** ± 1.29<br>0.96	5.05** ± 2.17<br>0.97	−5.08** ± 0.77<br>1.00	−12.29** ± 1.19<br>0.97	−18.99** ± 0.75<br>0.98	−0.64 ± 1.40<br>1.00	7.53** ± 1.35<br>1.00

Parameters (*N*)	**Calories, kcal**<br>%diff M ± SD<br>*r***	**Carb, g**<br>%diff M ± SD<br>*r***	**Protein, g**<br>%diff M ± SD<br>*r***	**Fat, g**<br>%diff M ± SD<br>*r***	**Sat Fat, g**<br>%diff M ± SD<br>*r***	**Cholesterol, mg**<br>%diff M ± SD<br>*r***	**Fiber, g**<br>%diff M ± SD<br>*r***
Chinese (10)	−9.74** ± 5.33<br>-0.08	12.34** ± 7.03<br>0.63	−9.44** ± 1.85<br>0.91	−32.50** ± 3.75<br>0.50	−34.13** ± 3.06<br>0.47	-12.59** ± 1.67<br>0.97	28.44** ± 7.26<br>0.84
Korean (13)	−4.47 ± 15.39<br>0.98	5.52 ± 21.72<br>0.98	−14.82** ± 12.96<br>0.98	−20.00** ± 8.39<br>0.99	−19.78** ± 12.14<br>0.99	−8.63** ± 7.49<br>0.99	13.73* ± 18.70<br>0.98
Smoothie-added (22)	−6.67 ± 25.56<br>0.66	−2.83 ± 28.95<br>0.50	−12.09* ± 21.17<br>0.72	−14.70** ± 22.68<br>0.81	−14.55** ± 21.76<br>0.83	−13.07** ± 20.14<br>0.83	−1.16 ± 34.39<br>0.16

Note: Carb: carbohydrate; Sat Fat: saturated fat; %diff: % difference against Food Frequency Questionnaire; M: mean; SD: standard deviation; *r*: correlation; **p <0.05*; ***p <0.001*.

Supplementary Table S2. Bias and agreement between Mobile Application and Food Frequency Questionnaire per domains of caloric ranges, energy nutrients, and various diets for vitamins Bs (*N*=135).

Parameters (*N*)	**Thiamin, mg**<br>%diff M ± SD<br>*r***	**Riboflavin, mg**<br>%diff M ± SD<br>*r***	**Niacin, mg**<br>%diff M ± SD<br>*r***	**Pyridoxine, mg**<br>%diff M ± SD<br>*r***	**Folate, mcg**<br>%diff M ± SD<br>*r***	**Cobalamin, mcg**<br>%diff M ± SD<br>*r***
Calories						
<1000 (54)	0.08 ± 15.37<br>0.65	−2.31 ± 16.36<br>0.37	0.87 ± 17.76<br>0.74	6.19* ± 20.58<br>0.71	2.06 ± 20.12<br>0.70	−21.02** ± 16.02<br>0.84
1000–2000 (63)	6.58** ± 11.13<br>0.89	0.60 ± 10.28<br>0.85	−0.02 ± 11.29<br>0.84	4.36** ± 9.85<br>0.98	7.04** ± 12.56<br>0.94	−14.87** ± 12.47<br>0.71
>2000 (18)	−4.81 ± 24.34<br>0.57	−8.31 ± 22.63<br>0.58	−9.40 ± 23.85<br>0.72	−9.11 ± 32.61<br>0.53	−6.53 ± 27.99<br>0.42	−26.31** ± 15.94<br>0.63
Energy Nutrients						
Fat	−13.91** ± 14.15<br>0.33	−22.38** ± 14.28<br>0.44	−11.85** ± 12.78<br>0.62	−14.25** ± 14.78<br>0.53	−14.84** ± 14.23<br>0.53	−17.51** ± 15.08<br>0.31
Protein	−5.16** ± 9.04<br>0.48	−14.91** ± 13.08<br>0.82	−5.62** ± 9.95<br>0.36	−6.17** ± 10.67<br>0.22	−5.00** ± 9.46<br>0.23	−13.84** ± 13.61<br>0.65
Carbohydrate	13.39** ± 16.35<br>0.79	−4.61* ± 18.30<br>0.80	14.81** ± 15.48<br>0.65	14.60** ± 15.81<br>0.80	14.24** ± 16.11<br>0.57	1.31 ± 20.67<br>0.47
Diet Type						
Pure Liquid (10)	8.62 ± 23.69<br>0.77	2.92 ± 29.51<br>−0.29	11.57 ± 34.19<br>0.67	22.70 ± 36.45<br>0.83	20.44 ± 35.87<br>0.67	−20.13* ± 25.65<br>0.50
Convenient Diet (30)	−0.38 ± 13.51<br>0.93	−2.01 ± 10.22<br>0.93	−6.56** ± 12.56<br>0.90	1.89 ± 10.32<br>0.79	0.76 ± 13.84<br>0.86	−9.82** ± 10.84<br>0.86
Canned Food (10)	−10.51** ± 8.02<br>0.89	−6.22** ± 3.70<br>0.78	−15.57** ± 1.92<br>0.93	4.19* ± 5.68<br>0.89	−3.11 ± 14.39<br>0.93	−9.95** ± 1.72<br>0.50
High School (10)	9.13** ± 4.78<br>0.95	0.47 ± 2.67<br>0.98	−0.24 ± 4.75<br>0.93	2.35 ± 4.01<br>0.90	4.51 ± 8.19<br>0.86	−15.43** ± 6.48<br>0.92
Fast Food (10)	0.24 ± 16.92<br>0.21	−0.27 ± 16.91<br>−0.01	−3.88 ± 18.43<br>-0.56	−0.86 ± 16.75<br>-0.56	0.87 ± 17.63<br>0.47	−4.07 ± 16.18<br>0.01
Ethnic Food (73)	4.57** ± 11.84<br>0.94	−1.38 ± 10.51<br>0.95	1.29 ± 9.21<br>0.94	3.21* ± 11.37<br>0.95	4.11** ± 12.80<br>0.93	−19.96** ± 11.24<br>0.90
Western Diet (40)	5.40** ± 11.41<br>0.86	0.63 ± 11.67<br>0.80	2.12 ± 8.89<br>0.79	1.80 ± 10.38<br>0.80	3.64 ± 13.74<br>0.79	−16.46** ± 11.64<br>0.86
American (10)	7.77 ± 13.11<br>0.52	4.54 ± 15.75<br>0.41	1.84 ± 6.91<br>0.57	3.71 ± 16.80<br>0.85	5.43 ± 17.35<br>0.78	−9.73** ± 6.53<br>0.62
Mexican (10)	13.03** ± 8.77<br>0.97	4.01 ± 7.07<br>0.75	0.59 ± 5.28<br>0.97	4.29 ± 6.89<br>0.29	11.42* ± 13.22<br>0.75	−12.92** ± 4.78<br>-0.15
Italian (10)	7.08** ± 2.24<br>0.92	−0.51 ± 5.20<br>0.39	9.47* ± 11.30<br>−0.09	2.95 ± 5.32<br>0.77	7.29** ± 1.77<br>0.97	−15.71** ± 9.93<br>-0.10

Parameters (*N*)	Thiamin, mg<br>%diff M ± SD<br>*r***	Riboflavin, mg<br>%diff M ± SD<br>*r***	Niacin, mg<br>%diff M ± SD<br>*r***	Pyridoxine, mg<br>%diff M ± SD<br>*r***	Folate, mcg<br>%diff M ± SD<br>*r***	Cobalamin, mcg<br>%diff M ± SD<br>*r***
Mediterranean (10)	−6.26 ± 9.23<br>0.45	−5.50 ± 13.86<br>−0.31	−3.41 ± 6.50<br>0.72	−3.76 ± 7.84<br>0.73	−9.60** ± 7.61<br>0.72	−27.48** ± 14.94<br>0.03
Eastern Diet (33)	3.56 ± 12.44<br>0.97	−3.81* ± 8.46<br>0.99	0.28 ± 9.63<br>0.98	4.92* ± 12.41<br>0.97	4.68* ± 11.74<br>0.97	−24.20** ± 9.24<br>0.96
Japanese (10)	−2.37** ± 2.24<br>0.95	−6.50** ± 2.75<br>0.84	−0.40 ± 2.12<br>0.98	−1.90* ± 2.40<br>0.98	−2.25** ± 1.14<br>1.00	−16.76** ± 0.43<br>1.00
Chinese (10)	15.09** ± 3.66<br>0.72	1.26 ± 3.77<br>0.66	−0.07 ± 2.70<br>0.78	7.31** ± 5.28<br>0.84	15.64** ± 2.62<br>0.94	−20.75** ± 0.84<br>1.00
Korean (13)	−0.75 ± 15.44<br>0.98	−5.65 ± 11.97<br>0.99	1.08 ± 15.41<br>0.98	8.33 ± 18.14<br>0.98	1.57 ± 14.52<br>0.98	−32.58** ± 9.74<br>0.98
Smoothie-added (22)	−3.47 ± 22.29<br>0.61	−4.76 ± 23.32<br>0.62	−6.19 ± 23.00<br>0.72	−3.33 ± 31.40<br>0.38	−4.09 ± 25.77<br>0.28	−26.94** ± 18.78<br>0.74

Note: %diff: % difference against Food Frequency Questionnaire; M: mean; SD: standard deviation; *r*: correlation; **p <0.05; ***p <0.001.*

Supplementary Table S3. Bias and agreement between Mobile Application and Food Frequency Questionnaire per domains of caloric ranges, energy nutrients, and various diets for methyl donors and vitamins A, C, and D (*N*=135).

Parameters (*N*)	Methionine^, g<br>%diff M ± SD<br>*r***	Choline, mg<br>%diff M ± SD<br>*r***	Glycine, g<br>%diff M ± SD<br>*r***	Vitamin A, IU<br>%diff M ± SD<br>*r***	Vitamin C, mcg<br>%diff M ± SD<br>*r***	Vitamin D, mcg<br>%diff M ± SD<br>*r***
Caloric Ranges						
<1000 (54)	−12.28** ± 12.87<br>0.86	-4.80* ± 15.77<br>0.72	-12.11** ± 15.60<br>0.84	39.41** ± 18.09<br>0.80	26.44** ± 31.94<br>0.85	−5.19** ± 10.87<br>0.94
1000–2000 (63)	−9.77** ± 9.64<br>0.90	-4.70** ± 10.10<br>0.91	-10.08** ± 10.27<br>0.88	42.57** ± 15.95<br>0.98	18.63** ± 19.36<br>0.99	−5.72** ± 13.36<br>0.90
>2000 (18)	−18.18** ± 20.09<br>0.75	-15.90* ± 24.77<br>0.61	-19.34** ± 21.32<br>0.75	−3.32 ± 55.10<br>0.55	−7.54 ± 40.76<br>0.56	−10.74* ± 15.76<br>0.52
Energy Nutrients						
Fat	−24.06** ± 13.93<br>0.47	-22.95** ± 13.46<br>0.60	-24.42** ± 14.20<br>0.52	−20.55** ± 14.74<br>0.44	−17.46** ± 14.73<br>0.34	−20.85** ± 14.11<br>0.27
Protein	−16.82** ± 11.97<br>0.90	-16.04** ± 11.76<br>0.93	-16.61** ± 11.85<br>0.91	−8.21** ± 9.10<br>0.38	−7.80** ± 10.15<br>0.45	−13.94** ± 13.16<br>0.48
Carbohydrate	−3.74 ± 19.46<br>0.71	-3.91 ± 18.78<br>0.85	-2.92 ± 19.49<br>0.79	9.85** ± 17.53<br>0.22	11.98** ± 16.88<br>0.70	−1.10 ± 19.19<br>0.48
Diet Type						
Pure Liquid (10)	−9.55 ± 21.34<br>0.62	7.91 ± 29.49<br>0.28	0.28 ± 25.91<br>0.63	50.90** ± 19.71<br>0.92	53.58** ± 39.23<br>0.89	−11.51 ± 19.67<br>0.73
Convenient Diet (30)	−12.93** ± 12.02<br>0.94	−6.45** ± 10.77<br>0.92	−14.27** ± 12.02<br>0.91	46.18** ± 9.02<br>0.92	19.36** ± 15.97<br>0.97	−1.92 ± 10.99<br>0.86
Canned Food (10)	−18.53** ± 3.84<br>0.98	−2.87 ± 7.73<br>0.90	−17.04** ± 7.48<br>0.99	37.52** ± 6.29<br>0.99	27.47** ± 19.59<br>0.98	−7.76** ± 1.12<br>0.99
High School (10)	−10.99** ± 5.16<br>0.93	−9.13** ± 3.36<br>0.93	−11.52** ± 5.97<br>0.92	47.22** ± 4.28<br>0.80	11.70** ± 6.53<br>0.87	−0.72 ± 4.45<br>0.83
Fast Food (10)	−9.28 ± 19.24<br>−0.14	−7.34 ± 16.73<br>0.34	−14.26* ± 18.89<br>−0.25	53.80** ± 7.43<br>-0.06	18.92** ± 16.02<br>0.99	2.72 ± 17.45<br>0.22
Ethnic Diet (73)	−11.31** ± 8.60<br>0.95	−7.34** ± 7.74<br>0.96	−12.47** ± 9.35<br>0.94	38.86** ± 14.99<br>0.94	19.00** ± 25.65<br>0.90	−4.90** ± 7.76<br>0.97
Western Diet (40)	−10.69** ± 8.16<br>0.83	−6.93** ± 8.41<br>0.80	−11.67** ± 8.33<br>0.85	38.72** ± 17.49<br>0.74	10.41** ± 21.37<br>0.88	−4.01** ± 9.03<br>0.91
American (10)	−6.60** ± 5.19<br>0.77	−2.69 ± 10.90<br>0.38	−8.10** ± 4.35<br>0.87	49.39** ± 14.59<br>0.90	4.98 ± 34.90<br>0.90	0.15 ± 9.49<br>0.51
Mexican (10)	−12.10** ± 3.11	−5.77** ± 5.44	−8.46** ± 4.24	49.23** ± 5.20	18.60** ± 16.10	−5.27 ± 9.81

Parameters (*N*)	Methionine^, g	Choline, mg	Glycine, g	Vitamin A, IU	Vitamin C, mcg	Vitamin D, mcg
	%diff M ± SD	%diff M ± SD	%diff M ± SD	%diff M ± SD	%diff M ± SD	%diff M ± SD
	*r***	*r***	*r***	*r***	*r***	*r***
	0.92	0.64	0.83	0.94	0.42	0.34
Italian (10)	−6.67* ± 7.72	−6.96** ± 6.16	−8.66* ± 9.46	40.45** ± 8.47	14.75** ± 11.07	−8.02* ± 9.30
	-0.10	-0.04	-0.15	0.93	0.98	0.39
Mediterranean (10)	−17.38** ± 10.18	−12.30** ± 8.17	−21.48** ± 5.66	15.81** ± 14.02	3.29 ± 13.94	−2.91 ± 6.34
	0.64	0.19	0.85	0.99	0.98	−0.03
Eastern Diet (33)	−12.06** ± 9.17	−7.83** ± 6.92	−13.44** ± 10.50	41.24** ± 11.36	29.42** ± 26.82	−5.99** ± 5.83
	0.97	0.98	0.96	0.96	0.92	1.00
Japanese (10)	−5.99** ± 0.57	−5.89** ± 1.10	−5.83** ± 0.50	36.14** ± 2.98	17.73** ± 8.79	−4.42** ± 0.36
	1.00	1.00	1.00	1.00	1.00	1.00
Chinese (10)	−12.69** ± 1.29	−6.26** ± 2.39	−14.19** ± 1.44	46.94** ± 3.45	37.56** ± 13.68	−5.7** ± 0.49
	0.98	0.85	0.96	0.99	0.92	1.00
Korean (13)	−16.24** ± 13.15	−10.53** ± 10.47	−18.72** ± 14.60	40.79** ± 16.72	32.15* ± 39.28	−7.41* ± 9.27
	0.97	0.99	0.97	0. 96	0.92	0.99
Smoothie-added (22)	−13.52** ± 20.28	−8.71 ± 25.96	−13.70** ± 21.92	−5.75 ± 46.33	−1.72 ± 38.54	−13.78** ± 20.07
	0.77	0.56	0.75	0.38	0.45	0.83

Note: ^Methionine RDI derived from a standard weight of 65 kg; %diff: % difference against Food Frequency Questionnaire; M: mean; SD: standard deviation; *r*: correlation;**p* <0.05; ***p* <0.001..

Supplementary Table S4. Bias and agreement between Mobile Application and Food Frequency Questionnaire per domains of caloric ranges, energy nutrients, and various diets for vitamin E and minerals (*N*=135).

**Parameters (*N*)**	**Vitamin E, mcg** %diff M ± SD *r***	**Zinc, mg** %diff M ± SD *r***	**Calcium, mg** %diff M ± SD *r***	**Magnesium, mg** %diff M ± SD *r***	**Iron, mg** %diff M ± SD *r***	**Sodium, mg** %diff M ± SD *r***
Caloric Ranges						
<1000 (54)	−30.12** ± 24.62	−12.27** ± 16.34	6.62 ± 44.59	5.38* ± 16.26	0.77 ± 21.29	−11.27** ± 18.16
	0.39	0.51	−0.09	0.65	0.70	0.88
1000–2000 (63)	−34.53** ± 10.94	−6.74** ± 11.61	−7.12** ± 12.76	5.69** ± 11.70	5.22** ± 9.46	−9.52* ± 29.56
	0.91	0.62	0.80	0.97	0.87	0.68
>2000 (18)	−35.14** ± 21.54	−17.90** ± 20.46	−14.91** ± 20.91	−8.19** ± 29.17	−9.63 ± 21.63	−27.37** ± 20.35
	0.53	0.65	0.51	0.54	0.67	0.66
Energy Nutrients						
Fat	−25.25** ± 13.55	−19.34** ± 12.97	−20.01** ± 13.90	−14.07** ± 15.15	−15.34** ± 14.74	−18.93** ± 17.31
	0.26	0.59	0.37	0.51	0.62	0.34
Protein	−14.26** ± 13.02	−13.81** ± 13.30	−13.37** ± 13.32	−4.95** ± 9.90	−5.08** ± 9.27	−13.23** ± 13.16
	0.42	0.86	0.68	0.38	0.32	0.49
Carbohydrate	−3.52 ± 20.55	−1.01 ± 19.72	−3.69 ± 18.13	14.70** ± 15.89	11.99** ± 17.12	0.84 ± 23.75
	0.48	0.78	0.67	0.81	0.58	0.44
Diet Type						
Pure Liquid (10)	−24.90 ± 56.06	3.54 ± 23.37	−11.03 ± 30.14	10.55 ± 29.75	22.64 ± 37.02	4.26 ± 33.72
	0.29	0.79	0.16	0.46	0.76	0.94
Convenient Diet (30)	−39.05** ± 10.31	−1.73 ± 11.20	−5.76** ± 10.77	4.71* ± 11.28	3.79 ± 10.82	−12.16** ± 13.80
	0.89	0.85	0.95	0.84	0.86	0.94
Canned Food (10)	−33.66** ± 10.61	3.41 ± 5.35	−10.02** ± 4.02	7.75* ± 9.18	5.54* ± 7.07	−21.94** ± 7.23
	0.99	0.97	0.87	0.96	0.95	0.98
High School (10)	−35.47** ± 5.45	−8.83** ± 3.21	−3.26 ± 5.05	4.85** ± 2.99	4.00* ± 4.32	−1.20 ± 7.16
	0.80	0.98	0.98	0.95	0.95	0.88
Fast Food (10)	−48.01** ± 8.05	0.24 ± 16.60	−4.00 ± 17.38	1.53 ± 17.18	1.83 ± 17.35	−13.33* ± 16.38
	-0.35	−0.31	0.20	−0.06	−0.26	-0.07
Ethnic Diet (73)	−31.50** ± 9.33	−14.75** ± 10.65	−3.13 ± 38.16	4.55** ± 11.83	0.27 ± 11.60	−11.82** ± 8.05
	0.89	0.92	0.29	0.96	0.95	0.95
Western Diet (40)	−32.25** ± 9.72	−13.44** ± 10.86	−11.61** ± 15.17	1.95 ± 12.12	0.78 ± 12.81	−8.12** ± 6.80
	0.65	0.77	0.54	0.57	0.87	0.94
American (10)	−42.43** ± 9.56	−14.44** ± 11.39	−18.97** ± 17.29	3.14 ± 19.73	3.29 ± 10.84	−6.85* ± 8.40
	0.79	0.07	−0.03	0.42	0.24	0.12
Mexican (10)	−35.34** ± 4.38	−9.76** ± 5.28	−11.21** ± 10.72	4.16 ± 10.00	5.93* ± 8.11	−6.69** ± 6.28
	0.54	0.74	0.72	0.51	0.91	0.85
Italian (10)	−21.58** ± 2.28	−9.29* ± 11.48	−11.18** ± 6.44	1.82 ± 5.23	9.90** ± 4.32	−9.83** ± 3.06
	0.96	−0.27	0.35	0.56	0.52	0.74
Mediterranean (10)	−29.63** ± 5.83	−20.25** ± 11.65	−5.07 ± 20.82	−1.31 ± 10.05	−16.00** ± 8.18	−9.12** ± 8.54
	0.92	−0.19	−0.16	0.40	0.49	0.19
Eastern Diet (33)	−30.60** ± 8.89	−16.34** ± 10.33	21.00* ± 48.94	7.71** ± 10.83	−0.34 ± 10.11	−16.29 ± 7.19
	0.95	0.96	0.40	0.99	0.98	0.98
Japanese (10)	−21.98** ± 1.94	−19.89** ± 5.65	−5.47** ± 1.49	3.72** ± 1.04	−1.42* ± 1.93	−17.48** ± 2.73
	0.98	0.84	0.96	0.99	0.91	0.88
Chinese (10)	−39.23** ± 7.25	−12.45** ± 3.48	1.33 ± 2.71	12.92** ± 5.28	6.91** ± 3.00	−10.69 ± 2.10
	0.68	0.39	0.87	0.63	0.68	0.90
Korean (13)	−30.58** ± 6.66	−16.59** ± 15.11	56.48** ± 64.10	6.76 ± 15.95	−5.08 ± 13.93	−19.69** ± 9.44
	0.99	0.97	0.25	0.99	0.99	0.99
Smoothie-added (22)	−32.46** ± 20.18	−14.38** ± 20.74	−13.58** ± 20.62	−3.52 ± 28.00	−7.40 ± 20.42	−33.00** ± 16.46
	0.51	0.69	0.58	0.30	0.67	0.77

Note: %diff: % difference against Food Frequency Questionnaire; M: mean; SD: standard deviation; *r*: correlation; **p <0.05; ****p <0.001.*

Supplementary Table S5. Progression on selecting significant factors contributing to the differences between Mobile Application and Food Frequency Questionnaire on total calories.

Parameters	Logistic Regression original model				Generalized Regression Elastic Net model validation			
	p (χ^2)	MR	AICc	AUC	p (χ^2)	MR	AICc	AUC
Caloric Ranges								
1000–2000	0.0122	0.2727	31.73	0.7521	0.0122	0.2727	31.73	0.7521
Energy Nutrients, % Difference								
Fat	0.7318	0.1818	21.50	0.9573	<0.0001	0.1818	21.50	0.9573
Carbohydrate	0.7267				<0.0001			
Protein	0.0477				0.0476			
Diet Types								
Pure liquid	0.0165	0.1364	33.04	0.8632	0.0164	0.1364	33.05	0.8632
Korean	0.0373				0.0375			
Japanese	0.8769				<0.0001			
Smoothie added	0.0086				0.0087			
Combined Factor Model: 3 Factors (Table 3)								
1000–2000 caloric range	0.0003	0.0455	18.18	0.9957	<0.0001	0.0455	18.19	0.9957
Carbohydrate % Difference	<0.0001				<0.0001			
Protein % Difference	<0.0001				<0.0001			
Combined Factor Model: 4 Factors								
1000–2000 caloric range	0.0015	0.0455	19.93	1.0000	0.0002	0.0455	19.93	1.0000
Carbohydrate % Difference	<0.0001				<0.0001			
Protein % Difference	0.0001				<0.0001			
Japanese diet	0.8982				<0.0001			

Note: MR: Misclassification rate; AICc: Akaike's information criterion with corrections; AUC: Area under the curve

Supplementary Table S6. Progression on selecting significant factors contributing to the differences between Mobile Application and Food Frequency Questionnaire on folate.

Parameters	Logistic Regression original model				Generalized Regression Elastic Net model validation			
	p (χ^2)	MR	AICc	AUC	p (χ^2)	MR	AICc	AUC
Caloric Ranges								
1000–2000	0.0005	0.3846	40.11	0.5938	0.0140	0.3846	39.75	0.5938
Energy Nutrients, % Difference								
Calories	0.0482	0.3077	37.90	0.8688	0.0196	0.3077	37.90	0.8688
Carbohydrate	0.0066				0.0021			
Protein	0.0052				0.0027			
Fiber	<0.0001				<0.0001			
Diet Types								
Canned food	0.0465	0.2692	42.81	0.8344	0.0422	0.2692	42.73	0.8438
Fast Food	0.0465				0.0428			
Italian	0.8889				<0.0001			
Mediterranean	0.8876				<0.0001			
Chinese	0.8822				<0.0001			
Japanese	0.8799				<0.0001			
Combined Factor Model: 4 Factors (Table 4)								
1000–2000 caloric range	0.0053	0.1154	30.71	0.9125	0.0084	0.1154	30.73	0.9125
Carbohydrate % Difference	0.0006				0.0003			
Fiber % Difference	0.0008				0.0008			
Mediterranean diet	0.9001				<0.0001			
Combined Factor Model: 6 Factors								
1000–2000 caloric range	0.00095	0.1154	38.72	0.9094	0.0250	0.1154	38.32	0.9094
Carbohydrate % Difference	0.0095				0.0026			
Protein % Difference	0.0313				0.0348			
Fiber % Difference	0.0011				0.0026			
Mediterranean diet	0.9011				<0.0001			
Chinese diet	0.8982				<0.0001			

Note: MR: Misclassification rate; AICc: Akaike's information criterion with corrections; AUC: Area under the curve

Supplementary Table S7. Progression on selecting significant factors contributing to the differences between Mobile Application and Food Frequency Questionnaire on cobalamin.

Parameters	Logistic Regression original model				Generalized Regression Elastic Net model validation			
	p (χ^2)	MR	AICc	AUC	p (χ^2)	MR	AICc	AUC
Caloric Ranges								
1000–2000	0.0013	0.2727	31.57	0.7521	0.0013	0.2727	31.57	0.7521
Energy Nutrients, % Difference								
Fat	0.0181	0.4545	36.02	0.6838	0.0165	0.4545	36.02	0.6838
Protein	0.0008				0.0006			
Diet Types								
Canned food	0.9024	0.2273	46.94	0.8462	<0.0001	0.2273	46.32	0.8547
Fast food	0.9024				<0.0001			
Mediterranean	0.0080				0.0097			
Korean	0.8453				<0.0001			
Chinese	0.8587				<0.0001			
Japanese	0.0080				0.0118			
Smoothie added	0.0028				0.0298			
Combined Factor Model: 3 Factors (Table 5)								
1000–2000 caloric range	<0.0001	0.2727	35.84	0.7906	<0.0001	0.2727	35.78	0.7906
Protein % Difference	0.0094				0.0140			
Chinese diet	0.8718				<0.0001			
Combined Factor Model: 4 Factors								
1000–2000 caloric range	0.0006	0.2727	39.78	0.8205	0.0010	0.2727	38.30	0.8205
Fat % Difference	0.0176				0.0232			
Protein % Difference	0.0042				0.0005			
Chinese diet	0.8432				<0.0001			
Combined Factor Model: 5 Factors								
1000–2000 caloric range	0.0360	0.2727	40.87	0.8376	0.0304	0.2727	40.04	0.8291
Fat % Difference	0.0068				0.0007			
Protein % Difference	0.0056				0.0002			
Korean diet	0.9031				<0.0001			
Chinese diet	0.8797				<0.0001			

Note: MR: Misclassification rate; AICc: Akaike's information criterion with corrections; AUC: Area under the curve

Supplementary Table S8. Summary on significant factors contributing to the differences between Mobile Application and Food Frequency Questionnaire on major nutrients.

Parameters	Carbohydrate % Difference	Protein % Difference	Fat % Difference	Saturated Fat % Difference	Cholesterol % Difference	Fiber % Difference
Caloric Ranges						
1000–2000	-	0.0179	-	-	-	-
Energy Nutrients, % Difference						
Calories	<0.0001	<0.0001	0.0065	-	-	-
Carbohydrate	n/a	-	-	-	-	<0.0001
Protein	-	n/a	-	-	0.0444	-
Fat	<0.0001	0.0012	n/a	0.0002	-	-
Saturated Fat	-	-	<0.0001	n/a	<0.0001	-
Cholesterol	-	-	-	0.0016	n/a	-
Fiber	<0.0001	-	-	-	-	n/a
Diet Types						
Mediterranean	<0.0001	-	-	-	-	-
Japanese	-	-	<0.0001	0.0003	<0.0001	<0.0001
Chinese	-	-	-	-	-	<0.0001
MR	0.1364	0.2273	0.1364	0.0455	0.2273	0.1818
AICc	21.85	33.80	20.97	21.36	29.25	30.46
AUC	0.9777	0.7436	0.9500	0.9436	0.9018	0.8512

Note: MR: Misclassification rate; AICc: Akaike's information criterion with corrections; AUC: Area under the curve**.** Insignificant parameters are excluded: caloric ranges of <1000 and >2000, pure liquid diet, canned diet, high school diet, fast food diet, American diet, Mexican diet, Italian diet, Korean diet, and smoothie-added diet.

Supplementary Table S9. Summary on significant factors contributing to the differences between Mobile Application and Food Frequency Questionnaire on methyl-donors and co-factors.

Parameters	Thiamin % Difference	Riboflavin % Difference	Niacin % Difference	Pyridoxine % Difference	Choline % Difference	Glycine % Difference	Zinc % Difference
Caloric Ranges							
<1000	<0.0001	-	-	-	-	-	-
Energy Nutrients, % Difference							
Calories	-	-	<0.0001	0.0010	0.0009	-	-
Protein	-	<0.0001	-	-	<0.0001	<0.0001	<0.0001
Saturated Fat	0.0015	-	-	-	-	-	-
Fiber	<0.0001	0.0004	0.0001	<0.0001	-	-	-
Diet Types							
Canned-food	<0.0001	<0.0001	<0.0001	-	0.0085	-	<0.0001
Fast-food	-	-	-	-	-	-	<0.0001
Mexican	-	-	-	-	-	0.0469	-
Italian	-	-	<0.0001	-	-	-	-
Japanese	-	<0.0001	-	<0.0001	-	<0.0001	<0.0001
MR	0.1250	0.1154	0.2917	0.0400	0.1667	0.1000	0.1000
AICc	30.63	32.43	35.43	20.53	36.53	25.33	31.48
AUC	0.9593	0.9077	0.8185	0.9867	0.8467	0.9400	0.9211

Note. MR: Misclassification rate; AICc: Akaike's information criterion with corrections; AUC: Area under the curve. Insignificant parameters are excluded: caloric ranges of 1000 – 2000 and >2000, carbohydrate % difference, fat % difference, cholesterol % difference, pure liquid diet, High school diet, American diet, Mediterranean diet, Chinese diet, Korean diet, and smoothie-added.

Supplementary Table S10. Summary on significant factors contributing to the differences between Mobile Application and Food Frequency Questionnaire on other vitamins and minerals.

Parameters	Vitamin A % Difference	Vitamin C % Difference	Vitamin D % Difference	Vitamin E % Difference	Calcium % Difference	Magnesium % Difference	Iron % Difference	Sodium % Difference
Caloric Ranges								
<1000	-	0.0116	-	-	-	-	-	-
Energy Nutrients, % Difference								
Calories	-	-	-	-	-	-	0.0010	-
Carbohydrate	-	-	-	<0.0001	<0.0001	0.0003	-	-
Protein	-	-	0.0014	-	-	-	0.0019	0.0190
Fat	0.0019	-	0.0179	0.0407	-	0.0153	-	
Saturated Fat	0.0308	-	-	-	-	-	-	-
Cholesterol	-	-	-	0.0002	0.0119	0.0026	-	-
Fiber	-	<0.0001	-	-	-	<0.0001	<0.0001	0.0077
Diet Types								
Canned-food	-	<0.0001	<0.0001	-	-	-	-	-
High School	-	-	-	-	-	-	-	<0.0001
Fast-food	<0.0001	-	-	-	-	-	-	-
American	-	-	-	-	0.0044	-	-	-
Italian	-	-	-	-	<0.0001	-	<0.0001	-
Chinese	-	-	<0.0001	-	-	-	-	-
Smoothie-added	-	-	-	-	-	-	-	<0.0001
MR	0.2273	0.3636	0.3182	0.1818	0.3636	0.2727	0.2727	0.2273
AICc	29.62	33.65	38.64	31.88	39.86	32.90	39.49	31.95
AUC	0.8504	0.7917	0.7500	0.8875	0.7946	0.8917	0.7650	0.9573

Note. MR: Misclassification rate; AICc: Akaike's information criterion with corrections; AUC: Area under the curve. Insignificant parameters are excluded: caloric ranges of 1000 – 2000 and >2000, pure liquid diet, Mexican diet, Mediterranean diet, Japanese diet, and Korean diet.

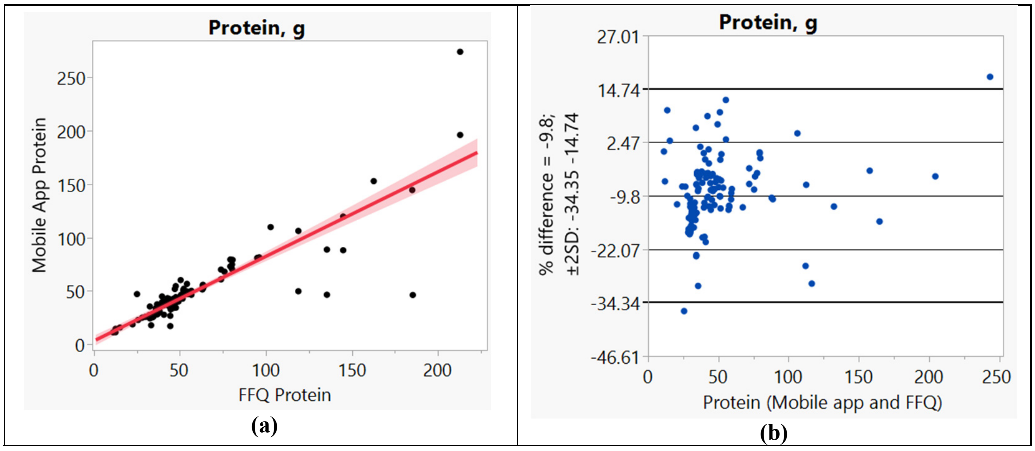

Supplementary Figure S1. (**a**) Correlation, (**b**) Bland and Altman plots between Mobile application and Food Frequency Questionnaire for protein.

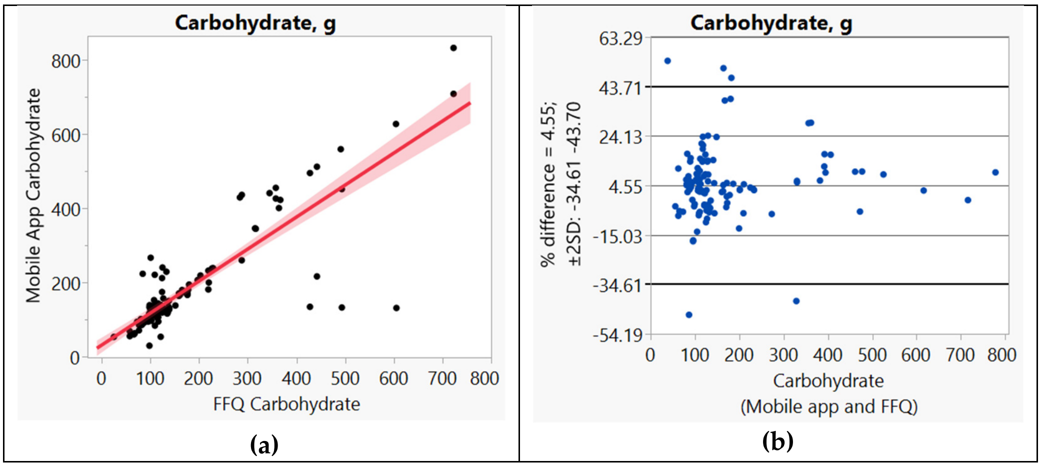

Supplementary Figure S2. (**a**) Correlation, (**b**) Bland and Altman plots between Mobile application and Food Frequency Questionnaire for carbohydrate.
